# Neurotoxicity evaluation of three root canal sealers on cultured rat trigeminal ganglion neurons

**DOI:** 10.4317/jced.52901

**Published:** 2017-01-01

**Authors:** Kursat Er, Ahmet Ayar, Omer-Faruk Kalkan, Sinan Canpolat, Tamer Tasdemir, Ulku Ozan

**Affiliations:** 1 DDS, PhD, Professor, Department of Endodontics, Faculty of Dentistry, Akdeniz University, Antalya, Turkey; 2 MDS, Professor, Department of Physiology, Faculty of Medicine, Karadeniz Technical University, Trabzon, Turkey; 3 Research Assistant, Department of Physiology, Faculty of Medicine, Karadeniz Technical University, Trabzon, Turkey; 4 MDS, Associate Professor, Department of Physiology, Faculty of Medicine, Firat University, Elazığ, Turkey; 5 DDS, PhD, Professor, Department of Endodontics, Faculty of Dentistry, Karadeniz Technical University, Trabzon, Turkey; 6 DDS, PhD, Assistant Professor, Department of Endodontics, Faculty of Dentistry, Abant İzzet Baysal University, Bolu, Turkey

## Abstract

**Background:**

The aim of this study was to investigate the possible neurotoxic effects of 3 root canal sealers (RCSs) (AH Plus, GuttaFlow, iRoot SP) on cultured rat trigeminal ganglion (TG) neurons.

**Material and Methods:**

Primary cultures of TG neurons were obtained from 1 to 2-day old rats. Freshly mixed RCSs were incubated in sterile phosphate buffered saline and cells were incubated with supernatants of the RCSs for different time intervals (1-, 3-, 6- and 24-h; 1 or 1/10 diluted) and viability/cytotoxicity was tested by counting the number of live cells. Pair of dishes with cells from the same culture incubated with only culture medium was considered as negative controls. Cell images were captured and acquired at x200 magnification using a microscope equipped with a camera using special image program. The viable cells were manually counted assigned from the images for each dose and incubation duration. Data was analysed by using 1-way analysis of variance with Tukey post hoc tests.

**Results:**

There was no significant change in cell viability after short duration of incubation (1- and 3-h) with the supernatant of any of RCSs, except for undiluted-AH Plus at 3-h. When AH Plus was compared with other RCSs, for diluted supernatants, there was only significant difference between iRoot SP and AH Plus at 24-h (*P*<0.05). Whereas undiluted-AH Plus was significantly more cytotoxic for 3-, 6- and 24-h periods as compared to respective incubation periods of undiluted other groups (*P*<0.05). GuttaFlow groups had similar neurotoxic effect on cells for all test periods.

**Conclusions:**

All tested RCSs exhibited a variable degree of neurotoxicity on these primary sensory neurons of orofacial tissues, depending on their chemical compositions. GuttaFlow and iRoot SP evoked a less toxic response to TG cells than AH Plus.

** Key words:**Neurotoxicity, trigeminal ganglia, cell culture, root canal sealer, AH Plus, GuttaFlow, iRoot SP.

## Introduction

Chemomechanical preparation of the root canal system is one of the major prerequisites of contemporary root canal treatment (RCT). During these procedures, dentin chips, remnants of pulp tissue, microorganisms, irrigants, intracanal dressings and/or filling materials may be extruded into the periradicular or neigboring tissues (e.g. maxillary sinus/mandibular canal) (1). Extrusion of these elements may cause undesired consequences ranging from inflammation to severe neurotoxicity (2).

Injury to the inferior alveolar nerve (IAN) is a relatively rare complication in dental practice (1). It may result in clinical sensory disorders such as pain, hyper/hypoaesthesia, anaesthesia, dysaesthesia, and paraesthesia (3).Most of the injury to the IAN is primarily iatrogenic. Inadequate (overextension and/or overfilling) RCT of the mandibular premolars and molars can damage the IAN bundle due to the proximity of the related roots (4-6). Additionally, the risk of this typeinjuryis dependent on several other factors, such as clinical tooth angulation, position of the anatomical foramen, the presence of accessory foramina, the presence/absence of cementum around the periapex, quality and density of the trabecular bone, and the degree of cortication of the IAN (6,7). RCT may also cause IAN injuries, which have been reported to occur in about 1% of mandibular premolar RCT (7) and about 10% of mandibular second molar RCT (6). The risk of this type of injury is also reported to be greater with the mandibular second molars compared with the mandibular premolars and first molar (8).

Therefore, necessary precautions should be taken during the RCT.

The use of root canal sealers is essential to promote the sealing ability of core material and to prevent the bacterial entry in complex root canals. Additionally, the biocompatibility of sealers is very important because they come into contact with periradicular tissues when compacting the filling core material and the tissue response to the sealers may influence the success of the RCT. In an attempt to find an ideal sealer, many materials have been developed based on the glass ionomer cement, zinc oxide eugenol, calcium hydroxide, epoxy/methacrylate resins, calcium silicate, and silicone for filling. Most of them have shown inadequate biological activity and have been exhibited a variable degree of toxicity depending on their chemical composition in studies (9,10).

To date, many studies (9-12) assessed the cytotoxicity of RCSs, although only a few authors observed the neurotoxic effects of extruded sealers (13-18). Common findings of these studies showed that all the tested sealers evoked variable degrees of neurotoxic responses to the tested cell cultures. Ahlgren *et al.* (19) reported that neurotoxic sealers cause changes in nerve membrane potential and transient or permanent block by inhibiting action potential conduction, which is the base of these sensory disorders. This *in vitro* study was designed to assess and compare the possible neurotoxicity of three sealers (AH Plus (an epoxy resin-based sealer, Dentsply De Trey, Konstanz, Germany), GuttaFlow (a silicone-based sealer, Colthane/Whaledent, Langenau, Germany), and iRoot SP (a calcium silicate-based sealer, Innovative Bioceramix, Vancouver, BC, Canada also known as EndoSequence BC Sealer, Brasseler, Savannah, GA, USA)) on cultured rat trigeminal ganglion (TG) neurons. The null hypothesis is that there is no significant difference in the neurotoxicity of all tested RCSs.

## Material and Methods

-Animals and rat TG primary culture

The study protocols were approved by the local Ethics Committee (protocol number AU 2013.09.04). Short-term primary cultures of TG neurons were obtained from 1 to 2-day old Wistar rats in aseptic conditions. Briefly, the animals were decapitated, the scalp and skull were cut, the brain was removed, both trigeminal ganglia were quickly harvested and temporarily collected in a petri dish filled with culture medium containing neurobasal A medium with B27 (Gibco Invitrogen, Paisley, UK), 5 mM glutamine, supplemented with antibiotics (Penicillin (5000 IU/mL)-Streptomycin (5000 mg/mL) (Gibco Invitrogen)). Afterward, the tissues were treated enzymatically with collagenase (0.125% in culture medium for 13 min at 37oC) (Sigma-Aldrich, Deisenhofen, Germany), followed by trypsin (0.25% in PBS for 6 minutes at 37oC) (Sigma-Aldrich). Then, the cells were mechanically dissociated by trituration with a fire polished glass pipette of decreasing tip diameter and after washing the cells were plated on poly-D-lysine/laminin coated round glass coverslips (Thermo Scientific, Menzel-Glaser, Braunschweig, Germany). Cells were maintained in the culture medium supplemented with nerve growth factor (NGF 2.5 S; Sigma-Aldrich) at 37°C in a 95% air/5% CO2 humidified incubator (Thermo Fisher Scientific Inc, Marietta, USA). Coverslips with cells were taken for neurotoxicity experiments from 3 h after plating up to 36h in culture.

-Preparation of supernatants of RCSs

Composition of the tested RCSs and their manufacturers were shown in  Table 1. Supernatants of RCSs were prepared according to the Al-Hiyasat *et al.* (11) RCSs were mixed according to the manufacturer’s instructions under aseptic conditions. One gram of each of the mixed materials was then dispensed into one well of a 6-well tissue culture plate. They were dispensed in the form of small discs so that the whole surface of the well of the tissue culture plate contained 20 discs of approximately the same size and weight (approximately 50 mg). The materials were covered with 10 mL of sterile phosphate buffered saline (PBS) and eluted for 1 week at 37°C. After 1 week, the plates were removed from the incubator and the supernatant was centrifuged at 750 ×g for 1 min to remove any solid particles. These supernatants were then used for neurotoxicity testing.

**Table 1:**
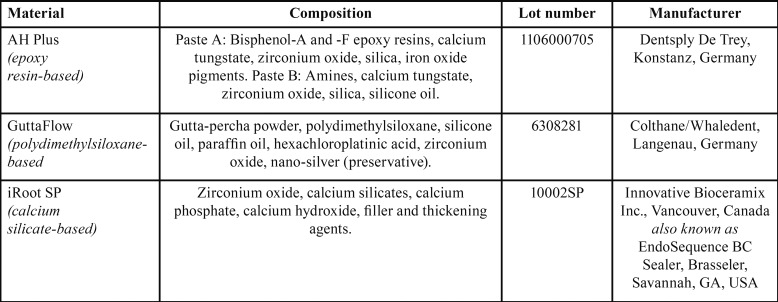
Composition and manufacturer of the test sealers.

-Cell number and neurotoxicity testing

Cultured TG neurones, routinely maintained in culture medium, were used in the experiments for cytotoxicity evaluation. Viability/cytotoxicity was tested by incubating the cells with concentrations (undiluted and diluted) of RCSs for different incubation time intervals (1-, 3-, 6- and 24-h). Cells were treated with culture medium containing either the undiluted or the diluted supernatant (1 in 10 v/v) of the sealers, and pair of dishes with cells from the same culture incubated with only culture medium were considered as negative controls.

For a typical experimental protocol, one dish of TG cells in culture medium served as control (treated with PBS as vehicle), and the second dish from the same cell culture was chosen to incubate with culture medium containing supernatants of RCS (either diluted or undiluted) for 1-, 3-, 6-, and 24-h, respectively.

Brightfield images of the cells were captured from multiple regions at x200 magnification and acquired through an inverted mi-croscope (Zeiss Axioobserver, Zeiss, Germany), equipped with a CCD camera (Cool-SNAP EZ; Roper Scientific, Tuscon, AZ, USA) using image acquisition system (VisiView imaging system, Visitron Systems, Germany). The extent of neurotoxicity was quantitated by manually counting the viable cells from the images. Off-line counting was performed by the author (OFK) who was blinded to the protocol of the image. Images were taken randomly for counting the cells. Numbers of cells from random microscopic fields were calculated by averaging the number of cells from at least two different experiments for each dose and incubation duration. Viable cell number was given as percentage of the untreated (vehicle treated) controls.

-Statistical analysis

Origin software package (Microcal, Northampton, USA) was employed for statistical analysis. Data are expressed as means ± standard error of mean (SEM). Statistical evaluations of differences between means of cellular death were evaluated using one-way ANOVA and Tukey post tests. Differences were considered significant at *P*<.05.

## Results

**Figure 1:**
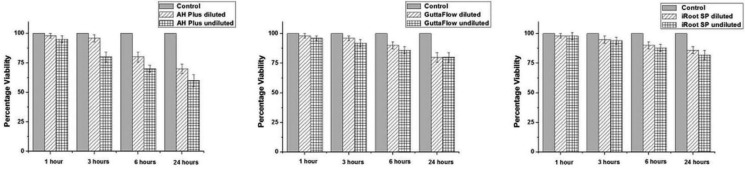
Time- and dose-dependent effects of tested RCSs on cultured rat TG neuron cell number and viability. Cells were incubated for 1, 3, 6 or 24 h in the presence of different sealers or in the absence of any sealers (control), as indicated. Bars represent means (with SD indicated) of three independent experiments. Viability of primary TG neurons was assessed by manual count of viable cells from at least 3 independent preparations, and expressed as mean percentage ± SD.

**Figure 2:**
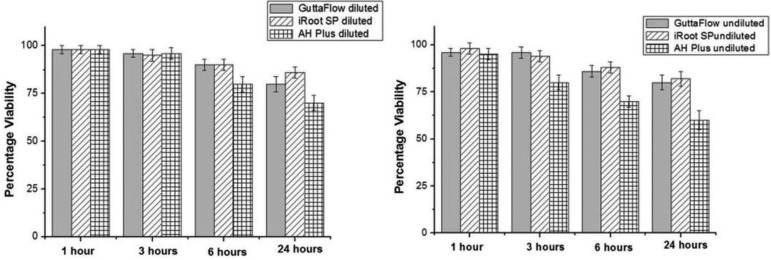
Graphical representation of the diluted and undiluted RCSs together.

**Figure 3:**
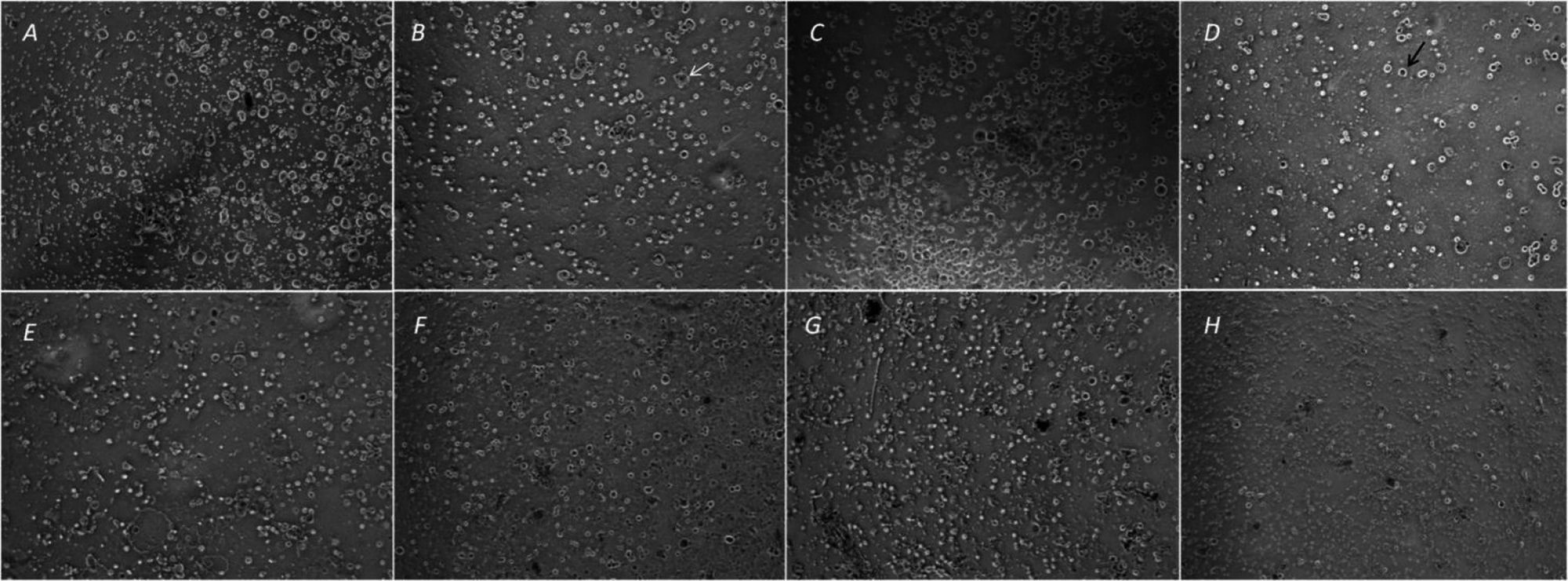
Microscopic image samples. A) control, 0-h. B) control, 24-h, arrows; green: a smaller-sized cell, red: a medium-sized cell, white: a large-sized cell. C) AH Plus, undilue, 1-h. D) AH Plus, dilue, 24-h, arrows; blue: a viable-cell, black: a dead cell, stained, it has been taken into the dye. E) Guttaflow, dilue, 3-h. F) GuttaFlow, undilue, 24-h. G) iRoot SP, dilue, 1-h. and H) iRoot SP, undilue, 24-h.

As shown in figure 1, incubation of the TG neurons with culture medium (controls) did not have any significant effect on cell viability for the test duration. There was no significant change in cell viability after short duration of incubation (1- and 3-h) with anyof the RCSs (AH Plus, GuttaFlow and iRoot SP; either diluted or undiluted supernatants), compared to respective control time points and their preincubation periods (*P*> 0.05), except for undiluted-AH Plus at 3-h (*P*< 0.05). Undiluted-AH Plus had significant reduction in percentage survival at 3-h (80±4% of preincubation period, *P*<0.05, Figs. 1-3). GuttaFlow (for both diluted and undiluted supernatants) had similar neurotoxic effect on cultured cells for all test time periods. For any of the incubation period tested, there was no significant difference between the GuttaFlow and iRoot SP, either diluted or undiluted (*P*> 0.05). When the AH Plus was compared with the other RCSs, for diluted supernatants there was only significant difference between iRoot SP and AH Plus for 24-h (*P*< 0.05) (Fig. 2), the rest has comparable level of neurotoxicity. Whereas undiluted-AH Plus was significantly more cytotoxic for 3-, 6- and 24-h incubation periods as compared to respective incubation periods of undiluted-GuttaFlow and undiluted-iRoot (*P* < 0.05) (Fig. 2). The signifcant neurotoxic effect of AH Plus groups were evident for 3-, 6- and 24-h incubation periods (*P*<0.05), except for diluted-AH Plus at 3-h (Figs. 1-3). The most cytotoxic effect was observed following incubation with undiluted-AH Plus. Undiluted-AH Plus caused 5±3%, 20±4%, 30±3% and 40±3% reductions in cell survival after 1-, 3-, 6- and 24-h incubation periods (Figs. 1,2). The respective values were 2±2%, 4±3%, 20±4% and 30±4% after 1-, 3-, 6- and 24-h incubation periods with diluted-AH Plus.

## Discussion

Extrusion of the sealers has been shown to have cyto-, geno-, and neurotoxic effects on the periradicular or close anatomical tissues (3). When the RCSs contacted with a nerve, it has been reported (20) that the sealers effect nerve transmission. Neurotoxicity of the sealers has been previously researched in some studies (13-18). For the first time, Brodin *et al.* (13) and Boisen & Brodin (14) compared the compound action potentials of certain sealers on rat phrenic nerve. Using the same technique, researchers (16) found that all tested sealers (Endomethasone, N2 Universal, Traitement SPAD, Sealapex, and CRCS) reversibly inhibit the compound action potential amplitudes on isolated rat sciatic nerves after local application (16). Asgari *et al.* (17) compared the action potential changes of AH 26 and Roth 801 sealers on snail F1 neurons and evaluated the behavior and electrical excitability of nerves after applying the sealer directly or in dissolved form to the solution where the nerve was placed. The results showed that AH 26 had a 2-way effect; its early phase effect is stimulating; however, later the sealer created significant inhibition in excitability and electrical behavior. A recent study (18) showed the sealers can directly activate trigeminal nociceptors, leading to a robust release of calcitonin gene-related peptide, and may therefore lead to pain and neurogenic inflammation.

The trigeminal nerve is a nerve responsible for sensation in the face and motor functions. The larger sensory part forms the ophthalmic, mandibular, and maxillary nerves that carry various senses from the skin, muscles, and joints of the face and mouth, as well as from the teeth. Most of these fibers originate from cells of the TG and project to the trigeminal nuclei in the brain stem (21). Various types of cells are found in the TG, including large-diameter, heavily myelinated Aα, Aβ and Aγ fibers associated with motor, proprioception, touch, pressure, and muscle spindle stretch functions. But it is the smaller, less myelinated Aδ and yet smaller and unmyelinated C fibers that conduct information likely to be perceived as pain (Fig. 3). Although no detailed subtype analysis was performed; large and medium-sized TG neurones were more affected than small-sized neurons in this study.

Neurotoxic effects of AH Plus, GuttaFlow and iRootSP RCSs was assessed on cultured rat TG neurons in this study. Our results showed that all tested sealers exhibited different levels of toxicity in different concentrations and times. No significant changes was observed in cell viability at 1- and 3-h time periods with any of the sealers, except for undiluted-AH Plus at 3-h. GuttaFlow and iRoot SP evoked a less toxic response to TG cells than AH Plus. The null hypothesis that there is no significant difference in the neurotoxicity of among the three sealers therefore has to be rejected. Among these sealers, an epoxy resin-based sealer AH Plus created a severe toxic irritation on cells. The diluted supernatants of sealers when compared with each other, there was only significant difference between iRoot SP and AH Plus at 24-h. However, undiluted-AH Plus was found more cytotoxic from the undiluted supernatants of GuttaFlow and iRoot at 3-, 6- and 24-h. Neurotoxic effect of AH Plus groups were evident for 3-, 6- and 24-h incubation periods, except for diluted-AH Plus at 3-h. It is not known which compound in AH Plus is the main causative element. The releases of formaldehyde or bisphenol A diglycidyl ether and amine reaction to initiate polymerization might explain the initial toxicity of this sealer (22). In addition, a cytotoxic by product appears later during stiffening.In studies (23,24), epoxy resin-based sealers have been shown to have significant cytotoxicity in the periradicular tissues by inducing inflammatory mediators (e.g. cyclooxygenase-2, nitric oxide synthase). Clinically, several case reports (4,5) demonstrated sensory loss on extrusion of AH Plus into the mandibular canal. There is in agreement with the mainly previous studies (9,25) that have documented the moderate to severe cytotoxic effect of AH Plus immediately after mixing and this initial toxicity decreases after stiffening. But, their studied time periods were vairous. For example, Pinna *et al.* (25)demonstrated the severe toxicity continued for the first 3-day and becames nontoxic after 3-week. The neurotoxicity of the tested sealers was evaluated after 1-, 3-, 6-, and 24-h time periods in this study. Contrary to these studies, toxicity of AH-Plus was increased with time.

GuttaFlowis arelatively new polydimethylsiloxane-based sealer used in root canal treatments. It was founded (26) that GuttaFlow possessed low genotoxicity. Besides, there are several studies (27,28) that report it to be nontoxic. Gencoglu *et al.* (29) using rats; malonyl aldehyde and glutathione levels in the tissue samples were evaluated. It showed that GuttaFlow exhibited good biocompatibility and acceptable tissue toxicity. In this study, we observed that diluted and undiluted supernatants of GuttaFlow displayed similar neurotoxic effect on cultured cells for all test time periods. When compared to AH Plus, it was found to have a significantly less toxic effect on cell viability and proliferation.

iRoot SP, another sealer tested, includes the concentrating and filling agents zirconium oxide, calcium silicates, calcium phosphate monobasic and calcium hydroxide. Mukthar-Fayyad (30) reported that it showed mild cytotoxicity in high concentrations and as the sealer was diluted the cytotoxicity was decreased. Toxic effect with higher concentrations was linked to its high pH and the calcium hydroxide release during stiffening. Contrary to this study, in other studies (9,12), it was reported that iRoot SP did not cause severe cytotoxic effects. According to our results, iRoot SP and GuttaFlow (for both diluted and undiluted supernatants) had similar neurotoxic effects on cultured cells for all test time periods.

As a result, all tested RCSs exhibited a variable degree of neurotoxicity on these primary sensory neurons of orofacial region, depending on their chemical compositions. GuttaFlow and iRoot SP evoked a less toxic response to TG cells than AH Plus. However, further animal and clinical studies are necessary to understand the overall behaviors of RCSs for succesful clinical applications.
